# *Chaetopterus* Luciferase: A Promising Tool for Online Lipid Peroxidation Detection

**DOI:** 10.3390/ijms262412119

**Published:** 2025-12-17

**Authors:** Alex S. Shcheglov, Konstantin V. Purtov, Renata I. Zagitova, Valery B. Kozhemyako, Alexandra S. Tsarkova, Astghik Pepoyan, Ilya V. Yampolsky

**Affiliations:** 1 Institute of Translational Medicine, Pirogov Russian National Research Medical University, Ostrovityanova 1, 117997 Moscow, Russia; 2 Shemyakin-Ovchinnikov Institute of Bioorganic Chemistry, Russian Academy of Sciences, Miklukho-Maklaya, 16/10, 117997 Moscow, Russia; 3 Institute of Biophysics SB RAS, Federal Research Center “Krasnoyarsk Science Center SB RAS”, Akademgorodok, 50/50, 660036 Krasnoyarsk, Russia; 4 Central Research Laboratory, Pacific State Medical University, Ostryakova 2, 690002 Vladivostok, Russia; 5 Scientific Research Institute of Food Science and Biotechnologies, Armenian National Agrarian University, Teryan 74, Yerevan 0009, Armenia

**Keywords:** ferroptosis, lipid peroxidation, luciferase, *Chaetopterus*, online detection

## Abstract

Lipid peroxidation plays a crucial role in living organisms. On the one hand, it contributes to the biosynthesis of several hormones; on the other, it can damage cellular structures, induce cell death, and participate in the pathogenesis of numerous human diseases. Therefore, the development of methods for real-time monitoring of lipid peroxidation, particularly within living systems, represents a highly relevant scientific goal. We previously demonstrated that peroxides of polyunsaturated fatty acids (PUFAs) or PUFA-containing lipids serve as substrates for *Chaetopterus* luciferase. Further studies revealed that the luminescence of this enzyme results from the decomposition products of PUFA peroxides or related lipids. Moreover, analogous luminescence-inducing products are generated during both enzymatic and chemical peroxidation of PUFAs or PUFA-containing lipids. Collectively, these findings indicate that *Chaetopterus* luciferase is a promising tool for online detection of lipid peroxidation.

## 1. Introduction

Lipid peroxidation is a free-radical chain reaction in which lipids containing unsaturated fatty acids react with molecular oxygen to form lipid peroxides. The biological significance of this process is immense. Fatty acid hydroperoxides serve as precursors for various animal and plant hormones, including prostaglandins, leukotrienes, jasmonic acid, and their derivatives [1]. However, uncontrolled lipid peroxidation leads to membrane damage, accumulation of toxic products, and ultimately cell death [2].

Recently, ferroptosis—a new form of programmed cell death characterized by iron-dependent membrane destruction via lipid peroxidation—was discovered [3]. Elevated lipid peroxidation levels are observed in multiple major diseases, such as myocardial infarction, atherosclerosis, ischemic injury, inflammation, neurodegeneration, and cancer [3]. Consequently, lipid peroxidation remains a central focus of modern biochemical and biomedical research.

Traditionally, lipid peroxidation levels are assessed using fluorescent probes that react with its specific products, including:

- Probes for lipid hydroperoxides (e.g., LiperFluo [4]);
- Probes for lipid radicals (e.g., α-parinaric acid [5], C11-BODIPY [6], and NBD-Pen [7]).

However, many of these probes exhibit cytotoxicity, limiting their use for real-time monitoring or in vivo imaging [8].

Bioluminescent systems offer a powerful alternative, combining high sensitivity, minimal background, and compatibility with live imaging. To date, around ten distinct luciferase-luciferin systems have been characterized, forming the basis for diverse biosensing and imaging applications [9], including in vivo visualization in laboratory animals [10].

We previously showed that *Chaetopterus* luciferase luminescence requires conjugated peroxides of polyunsaturated fatty acids and Fe^2+^ ions [11]. Here, we explore its potential as a bioluminescent reporter for real-time monitoring of lipid peroxidation.

## 2. Results

**Figure 1:**
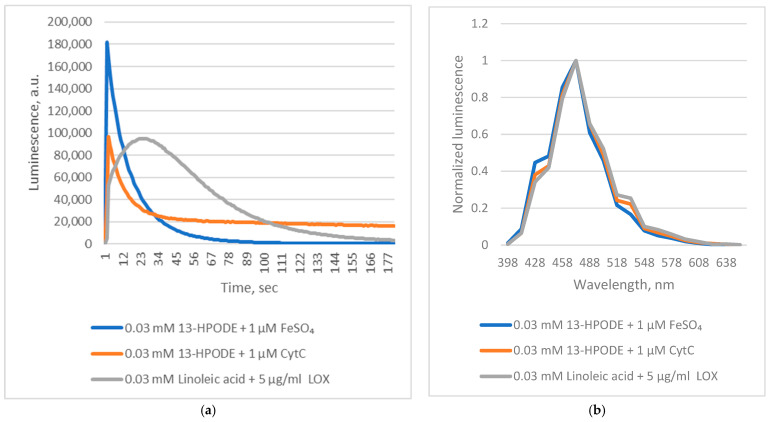
*Chaetopterus* bioluminescent reactions with linoleic acid and its peroxide. (**a**)—kinetics; (**b**)—spectrum.

Our earlier work demonstrated that *Chaetopterus* luciferase requires conjugated polyunsaturated fatty acid (PUFA) peroxides and Fe^2+^ ions for luminescence [11,12]. However, the precise role of these components remained unclear. Since fatty acid peroxides readily decompose in the presence of Fe^2+^, forming radical intermediates and secondary products [13,14], we hypothesized that Fe^2+^ could (1) act as a cofactor of luciferase, or (2) catalyze peroxide decomposition, generating the true substrates for the enzyme. To test this, we examined whether cytochrome C (Cyt C)—a heme-containing redox protein—could substitute Fe^2+^. Cyt C catalyzed luminescence with efficiency comparable to Fe^2+^ at equivalent concentrations (Figure 1). Importantly, the Fe^2+^ chelator phenanthroline, which completely inhibits iron-driven luminescence (unpublished data), did not affect the Cyt C-mediated reaction.

**Figure 2:**
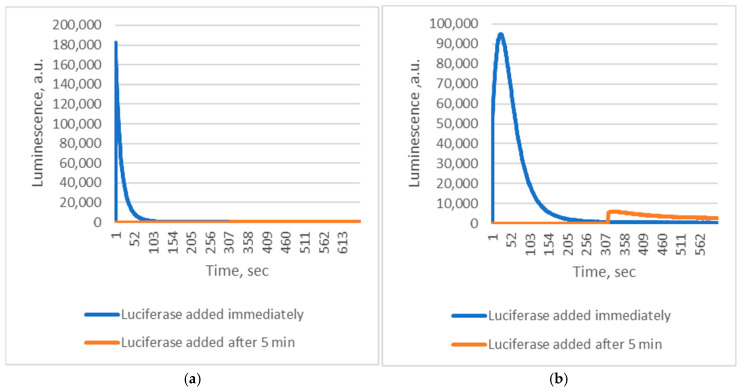
Kinetics of *Chaetopterus* bioluminescent reactions with different substrates. (**a**)—0.03 mM 13-HPODE + 1 µM FeSO_4_; (**b**)—0.03 mM linoleic acid + 5 µg/mL LOX.

These findings indicate that *Chaetopterus* luciferase does not require Fe^2+^ as a cofactor. Instead, luminescence results from the enzyme’s interaction with short-lived products generated during Fe^2+^-catalyzed decomposition of conjugated fatty acid peroxides. Reactive alkoxyl radicals are known to form during Fe^2+^-induced decomposition of fatty acid peroxides [14]. However, this non-enzymatic process produces a complex and poorly defined mixture of reactive species, complicating mechanistic studies. In contrast, enzymatic oxidation of PUFAs by lipoxygenases generates alkoxyl radicals and derivatives in a more controlled manner [15]. We therefore hypothesized that lipoxygenase-mediated oxidation of PUFAs could also yield substrates for *Chaetopterus* luciferase. Supporting this idea, we observed that soybean lipoxygenase (LOX)-mediated peroxidation of linoleic acid produced luminescence comparable in intensity to that obtained with pre-formed linoleic acid peroxide and Fe^2+^ (Figure 1), though with distinct kinetics. Phenanthroline did not inhibit the LOX-driven reaction, suggesting that enzymatically generated intermediates directly induce luminescence. When oxidation of linoleic acid (via LOX) or decomposition of its peroxide (via Fe^2+^) was initiated before adding luciferase, luminescence was at least 10 times weaker if the enzyme addition was delayed by five minutes (Figure 2). This confirms the extreme instability of the luminescent intermediates.

**Figure 3:**
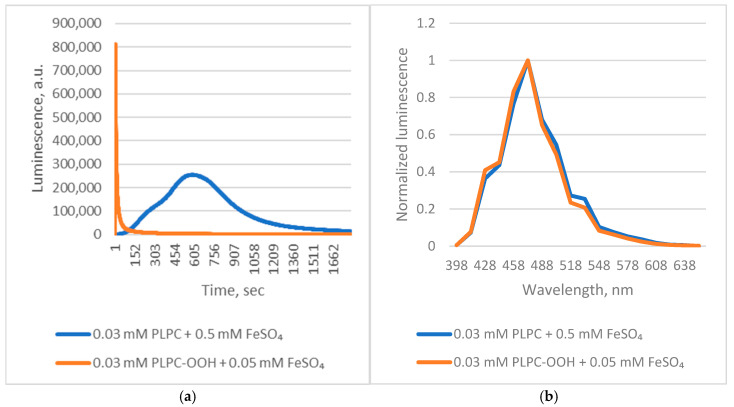
*Chaetopterus* bioluminescent reactions with PLPC and its peroxide. (**a**)—kinetics; (**b**)—spectrum.

Applying these insights, we examined *Chaetopterus* luciferase luminescence during Fe^2+^-induced peroxidation of native 1-palmitoyl-2-linoleoyl-sn-glycero-3-phosphocholine (PLPC). The reaction triggered strong luminescence with kinetic characteristics typical of autocatalytic lipid peroxidation, including a lag phase (Figure 3).

**Figure 4:**
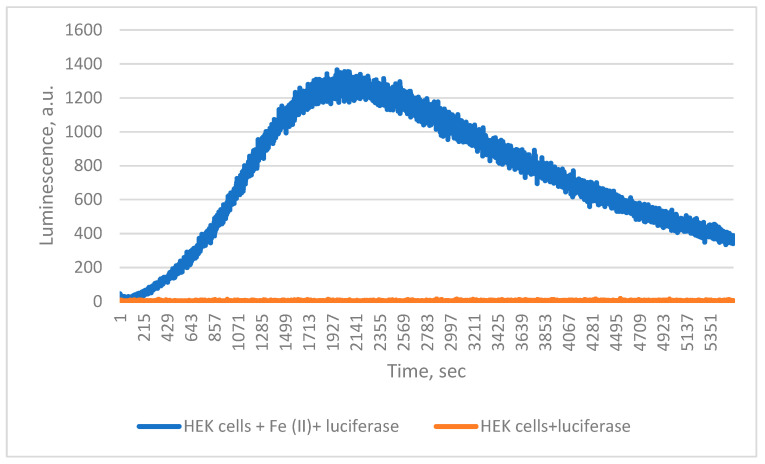
Detection of 0.5 mM FeSO_4_ induced lipid peroxidation in HEK cells by *Chaetopterus* luciferase.

Finally, when ferroptosis was simulated in HEK 293 T cells by Fe^2+^ treatment in the presence of *Chaetopterus* luciferase, luminescence increased steadily, unlike in untreated controls (Figure 4). During incubation, cell death approximately doubled, from 6% to 13%.

## 3. Discussion

Visualization of intracellular processes in live organisms remains a key scientific challenge. Several classes of probes are used for this purpose:

- Small molecules, often fluorescent, that bind specific structures or respond to environmental changes [16];
- Fluorescent proteins, including fusion constructs and engineered sensors [17];
- Bioluminescent systems, and synthetic sensors derived from them [18].

Small-molecule probes are primarily limited to cell culture due to high cost, poor penetration, and degradation. Fluorescent proteins overcome many of these limitations but may oligomerize and suffer from tissue autofluorescence interference.

Bioluminescent systems, in contrast, exhibit negligible background. Their main limitation is the need for luciferin supplementation. Recently, autonomous bioluminescent systems that function without external luciferin have gained attention [19].

Currently, only small-molecule sensors are available for detecting lipid peroxidation, highlighting the need for a genetically encodable alternative. Our results demonstrate that *Chaetopterus* luciferase utilizes short-lived byproducts of lipid peroxidation as substrates, making it a natural, genetically encoded bioluminescent sensor for lipid peroxidation that requires no exogenous luciferin.

It is known that active alkoxyl and peroxyl radicals are formed during chemical or enzymatic lipid peroxidation. Peroxyl radicals formed during Fe^3+^-mediated PUFAs-peroxide decomposition do not trigger *Chaetopterus* luminescence [12], suggesting that alkoxyl radicals or their derivatives serve as the actual substrates.

During ferroptosis, iron-dependent lipid peroxidation causes membrane destruction [3]. Our results show that *Chaetopterus* luciferase can detect such membrane damage, underscoring its potential as a tool for real-time monitoring of ferroptosis and oxidative stress.

## 4. Materials and Methods

Reagents. 1-Palmitoyl-2-linoleoyl-sn-glycero-3-phosphocholine (PLPC) was obtained from Avanti Polar Lipids (Alabaster, AL, USA). Linoleic acid was purchased from Merck KGaA (Darmstadt, Germany). Soybean lipoxygenase (Type I-B, ≥50,000 U/mg) and all other reagents were from Sigma-Aldrich (St. Louis, MO, USA), unless otherwise specified. 1-Palmitoyl-2-hydroperoxyoctadecadienoyl-sn-glycero-3-phosphocholine (PLPC-OOH) and (9Z,11E)-13-hydroperoxyoctadeca-9,11-dienoic acid (13-HPODE) were synthesized as described previously [11]. *Chaetopterus* luciferase was purified from natural sources (see Supplementary Material).

Biochemical assays. Reaction mixtures (100 µL) contained phosphate-buffered saline (PBS, pH 7.4), luciferase (1 µg, 1 µL of 1 mg/mL stock), and 3 mM methanolic substrate solution (1 µL). Initiators of peroxidation were added as follows: FeSO_4_ (0.1 mM, 1 µL) or cytochrome C (0.1 mM, 1 µL) for linoleic acid peroxide assays; soybean lipoxygenase (0.5 mg/mL, 1 µL) for linoleic acid; and FeSO_4_ (50 mM or 5 mM, 1 µL) for PLPC and PLPC-OOH assays, respectively. Where indicated, 1,10-phenanthroline was used at a final concentration of 0.1 mM. Luminescence was recorded immediately after mixing at room temperature.

Cell-based assays. HEK 293 T cells were grown in DMEM supplemented with 2 mM glutamine and 10% FBS (PanEko, Moscow, Russia) to monolayer confluence, detached with trypsin/versene, centrifuged (900 *g*, 5 min), and resuspended in PBS. Cell concentration and viability were determined using trypan blue and a Luna cell counter (Logos Biosystems, Gyeonggi-do, Republic of Korea). Reaction mixtures contained 50 µL PBS, 1 µL of 1 mg/mL luciferase, 1 µL of 50 mM FeSO_4_, and 5 × 10^5^ cells in 100 µL PBS. Luciferase was pre-incubated with FeSO_4_ for 2 min before cell addition. Luminescence was measured immediately at room temperature.

Instrumentation. Bioluminescence kinetics were recorded using a BLM-530 luminometer (Oberon-K, Krasnoyarsk, Russia) equipped with a Hamamatsu H12056P-110 photomultiplier (HAMAMATSU PHOTONICS K.K., Hamamatsu City, Japan) [20]. Bioluminescence spectrums were recorded using microplate reader Spark (Tecan, Grödig, Austria).

## Data Availability

The original contributions presented in this study are included in the article/Supplementary Material. Further inquiries can be directed to the corresponding author.

## Supplementary Materials

The following supporting information can be downloaded at: https://www.mdpi.com/article/10.3390/ijms262412119/s1.

## Abbreviations

The following abbreviations are used in this manuscript:

Cyt C	Cytochrome C
DMEM	Dulbecco’s modified Eagle’s medium
FBS	fetal bovine serum
HEK	Human Embryonic Kidney
13-HPODE	(9Z,11E)-13-hydroperoxyoctadecaenoic acid
LOX	soybean lipoxygenase, lipoxidase from Glycine max
PBS	phosphate-buffered saline pH 7.4
PLPC	1-palmitoyl-2-linoleoyl-sn-glycero-3-phosphocholine
PLPC-OOH	1-palmitoyl-2-hydroperoxyoctadecadienoyl-sn-glycero-3-phosphocholine
PUFAs	polyunsaturated fatty acids
