# Supplementary material for: Chaetopterus Luciferase: A Promising Tool for Online Lipid Peroxidation Detection
Source: Int J Mol Sci. 2025 Dec 17;26(24):12119. doi: 10.3390/ijms262412119 (PMC12733232; doi:10.3390/ijms262412119)
Supplement: Supplementary file 1 [file ijms-26-12119-s001.zip › ijms-4010832-supplementary.pdf]

Supporting information for

Chaetopterus Luciferase: A Promising Tool for Online Lipid Peroxidation Detection

Alex S. Shcheglov, Konstantin V. Purtov, Renata I. Zagitova, Valery B. Kozhemyako, Alexandra S. Tsarkova, Astghik Pepoyan, Ilya V. Yampolsky

Chaetopterus Luciferase purification

C. variopedatus was collected at Trinity bay in the Possiet Gulf of Japan Sea (Russia). A total of 100 g of the frozen *C. variopedatus* biomass were homogenized in 900 mL of 50 mM Tris buffer, pH 7.5. The homogenate was sonicated using Ultrasonic Disintegrator UD-20 (Techpan; Poland) 5 times for 2 min on ice and subsequently centrifuged (25 000 g x 20 min) at 4°C. Supernatant was supplemented with ammonium sulfate to the final concentration of 500 mM and passed through a cellulose DEAE column (Cellulose DEAE-32, Serva; Germany), equilibrated with 500 mM ammonium sulfate. The resulting filtrate was loaded onto the 25 x 100 mm Phenyl Sepharose CL-4B column (Cytiva; USA), equilibrated with 500 mM ammonium sulfate. Luciferase was eluted with 5 mM Tris-HCl buffer, pH 7.5.

Fractions, possessing luciferase activity, were combined and loaded onto the 16 x 200 mm Sepharose DEAE FF column (Cytiva; USA), equilibrated with 20 mM Tris-HCl, pH 7.5; the column was washed with the same buffer, and the linear gradient elution was performed. Buffer A: 20 mM Tris-HCl, pH 7.5; buffer B: 500 mM NaCl, 20 mM Tris-HCl, pH 7.5. The flow rate was 4 mL/min, and the time of gradient was 25 min.

The resulting preparation was concentrated on the 10 kDa cell (Amicon; Ireland) and loaded onto the 26 x 400 mm Sephacryl S200 column (Cytiva; USA), equilibrated with 200 mM NaCl and 20 mM Tris-HCl, pH 7.5. Elution was performed with the same buffer at a rate of 1.5 mL/min. The pooled fractions possessing luciferase activity were supplemented with ammonium sulfate to the final concentration of 500 mM, the resulting solution was loaded onto the 5 × 90 mm C8 column (Cytiva; USA). The linear gradient elution was carried out. Buffer A: 500 mM ammonium sulfate, 5 mM Tris-HCl, pH 7,5; buffer B: 5 mM Tris-HCl, pH 7.5. The flow rate was 0.5 mL/min, and the time of gradient was 80 min.

The resulting luciferase preparation was diluted twice with distilled water and loaded onto the 3 x 50 mm monoQ column (Cytiva; USA), equilibrated with 20 mM Tris-HCl, pH 7.5; the column was washed with the same buffer, and the linear gradient elution was performed. Buffer A: 20 mM Tris-HCl, pH 7.5; buffer B: 500 mM NaCl, 20 mM Tris-HCl, pH 7.5. The flow rate was 0.5 mL/min, and the time of gradient was 80 min.

The preparation was concentrated to a volume of 200 µL on the 10 kDa centrifuge filter (Amicon; Ireland) with subsequent gel filtration through the 10 x 300 mm Superdex 200 column (Cytiva; USA), equilibrated with 100 mM NaCl, 50 mM Tris-HCl, pH 7.5. Elution was performed with the same buffer at a rate of 0.8 mL/min. The maximum protein concentration in the fraction coincided with the maximum luciferase activity.

Chaetopterus Luciferase analysis

The molecular mass of *Chaetopterus* luciferase was determined using size-exclusion chromatography with a Superdex 200 Increase 5/150GL column. The column was pre-calibrated with a set of standard proteins of known molecular masses. The native molecular mass of luciferase, calculated from the gel filtration results, was approximately 68 kDa.

The resulting preparation was subsequently analyzed by SDS-PAGE (Figure S1, lane 3), revealing the presence of two proteins with approximate molecular masses of 14 and 17 kDa. Considering the native luciferase's mass of 68 kDa, it can be hypothesized that it exists as a tetramer.

To further investigate, the preparation was treated with the deglycosylating enzyme PNGase F (New England Biolabs) (Figure S1, lane 2) under denaturing conditions. This treatment demonstrated that the 17 kDa protein is the glycosylated form of the 14 kDa protein. The SDS-PAGE data suggest that the resulting preparation contains over 90% luciferase.

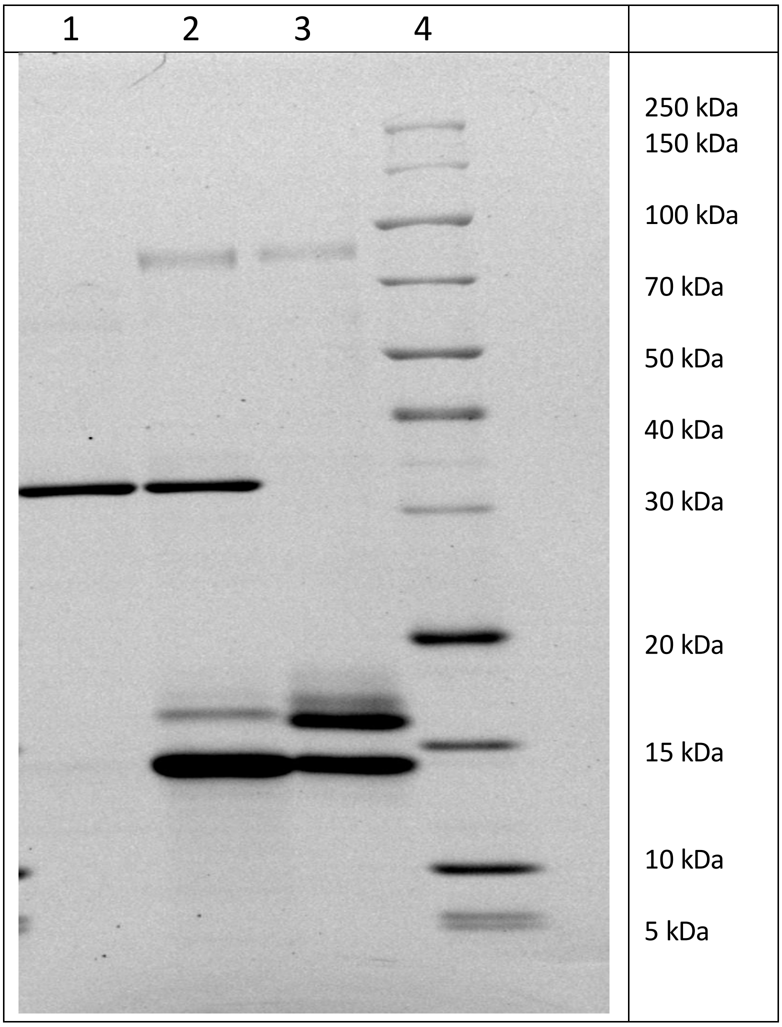

Figure S1. SDS-PAGE of *Chaetopterus* Luciferase.

Lane 1 PNGase F, Lane 2 *Chaetopterus* Luciferase+ PNGase F, Lane 3 *Chaetopterus* Luciferase, lane 4 PageRuler broad range Unstained Protein Ladder (Thermo Scientific).
